# The Interplay between NLRs and Autophagy in Immunity and Inflammation

**DOI:** 10.3389/fimmu.2013.00361

**Published:** 2013-11-11

**Authors:** Leticia A. M. Carneiro, Leonardo H. Travassos

**Affiliations:** ^1^Department of Immunology, Federal University of Rio de Janeiro, Rio de Janeiro, Brazil; ^2^Institute of Biophysics Carlos Chagas Filho, Federal University of Rio de Janeiro, Rio de Janeiro, Brazil

**Keywords:** NLR proteins, autophagy, inflammasomes, inflammation, infection, Crohn’s disease, innate immunity

## Abstract

Since they were first described as cytosolic sensors of microbial molecules a decade ago, the Nod-like receptors (NLRs) have been shown to have many different and important roles in various aspects of immune and inflammatory responses, ranging from antimicrobial mechanisms to control of adaptive responses. In this review, we focus on the interplay between NLRs and autophagy, an evolutionarily conserved mechanism that is crucial for homeostasis and has recently been shown to be involved in the protective response against infections. Furthermore, the association between mutations of NLRs as well as proteins that form the autophagic machinery and inflammatory diseases such as Crohn’s disease highlight the importance of these proteins and their interactions in the regulation of inflammation.

## Introduction

Homeostasis in multicellular organisms is dependent on the ability to detect and adapt to a myriad of environmental variations and insults, including exposure to microbes. Early detection of microbes is a crucial step in the defense strategy. Throughout evolution, the continuous interplay between multicellular organisms and microbes has led to the selection of sensors that allow early detection and initiation of the immune response against infections. This detection is based on the recognition of “microbial-associated molecular patterns” (MAMPs), which represent a signature of microbial origin, such as lipopolysaccharide (LPS), peptidoglycan (PG), flagellin, and nucleic acids from bacteria and viruses, or of “danger-associated molecular patterns” (DAMPs), which indicate the existence of cellular damage, such as extracellular ATP and HGMB1 by “pattern recognition molecules”(PRMs). Upon activation, PRMs trigger several protective responses that include the recruitment of phagocytic cells; secretion of chemokines, cytokines, and antimicrobial peptides; and priming of dendritic cells (DCs), engaging the adaptive immune system. Several families of PRMs have been described and include “Toll-like receptors” (TLRs), “RIG-I like receptors” (RLRs), “C-lectin type like receptors” (CLR), and “Nod-like receptors” (NLRs). In addition to their role in innate and adaptive immune responses, all of them have been recently implicated in the control of autophagy, an adaptive cellular response to environmental and microbial-induced stress. In this review, we highlight the role of NLR signaling in the control of autophagy and vice versa, the mechanisms involved and implications for inflammatory diseases such as Crohn’s disease (CD) and type 2 diabetes (T2D).

## NLR Proteins

Soon after the discovery of the transmembrane TLRs, it became evident that additional sensors were necessary for the surveillance of the cytosol. More than a decade ago, the demonstration that a mammalian homolog of plant disease-resistance (*R*) proteins called Nod1 could detect the presence of intracellular *Shigella flexneri* and activate the transcription factor nuclear factor κB (NF-κB) in epithelial cells *in vitro* inaugurated the studies on the role of NLRs as innate immune intracellular sensors (1). Subsequent studies have now set the number of human NLRs at approximately 20 and indicated their involvement in detecting not only microbial components but also DAMPs such as ATP, mitochondrial DNA (mtDNA) and reactive oxygen species (ROS) (2).

Due to the lack of signal peptides or transmembrane domains in their amino acid sequences, NLRs are thought to be exclusively located inside the cell. Both plant and animal NLRs are signal-transduction ATPases with numerous domains (STAND) P-loop ATPases of the AAA^+^ superfamily. The typical NLR protein contains the following domains: (a) a C-terminal leucine-rich repeat (LRR) domain, involved in sensing; (b) a central NATCH [Naip, CIITA, HET-E (plant het product involved in vegetative incompatibility)] and TP-1 (telomerase-associated protein 1 that mediates self-oligomerization and is essential for activation of NLRs); and (c) an N-terminal effector domain, responsible for protein–protein interactions with adapter molecules and signal transduction. Based on the nature of the N-terminal domains, NLRs have been separated into the NLRC subfamily, containing a CARD domain (caspase activation and recruitment domain); the NLRP subfamily, containing a pyrin domain; and the NAIP subfamily, which includes three (BIRs) baculovirus inhibitors of the apoptosis protein repeat domain (3).

## Nod1 (NLRC1) and Nod2 (NLRC2) are Intracellular Peptidoglycan Sensors

Nod1 and Nod2 were the first NLRs identified as MAMP detectors when two concomitant studies demonstrated that Nod2 detects muramyl-dipeptide (MDP), a common motif found in Gram-negative and Gram-positive PG and a major component of adjuvants (1, 4–6). Nod1, in contrast, recognizes PG containing the minimal motif *meso*-diaminopimelic acid (DAP), an amino acid found in Gram-negative and some Gram-positive bacteria, such as *Listeria monocytogenes* and *Bacillus subtilis*. The naturally occurring PG moieties sensed by human and mouse Nod1 are GlcNAc-MurNAc-l-Ala-d-Glu-*meso*-DAP (GM-triDAP) and GlcNAc-MurNAc-l-Ala-d-Glu-*meso*-DAP-d-Ala (GM-tetraDAP), respectively. After additional studies demonstrated that PG recognition by TLR2 was due to contaminants commonly found in PG preparations, it became clear that Nod1 and Nod2 are the only known PG sensors (2, 7–9). The NLR ligands/activators are summarized in Table 1.

**Table 1:** **NLR proteins involved in autophagy and their activators**.

NLR protein	Activator	Reference
Nod1 (NLRC1)	FK156 (d-lactyl-l-Ala-γ-Glu-*meso*-DAP)	Uehara et al. (10) and Magalhaes et al. (11)
	FK565 (Heptanoly)	Uehara et al. (10)
	*Meso*-lanthionine, *meso*-DAP	Uehara et al. (12)
	iEDAP (γ-d-Glu-*meso*-DAP)	Girardin et al. (159) and Chamaillard et al. (9)
	TriDAP (l-Ala-γ-d-Glu-meso-DAP)	Girardin et al. (4)
	TCT (GlcNAc-(anhydro) MurNAc-l-Ala-γ-d-Glu-mesoDAP-d-Ala)	Magalhaes et al. (11)
	*Bacillus* species	Hasegawa et al. (13)
	*L. pneumophila*	Hasegawa et al. (13)
	*S. typhimurium*	Hasegawa et al. (13) and Le Bourhis et al. (14)
	*H. pylori*	Viala et al. (15)
	*Pseudomonas* species	Travassos et al. (7)
	*Chlamydia* species	Kavathas et al. (16), Buchholz and Stephens (17), Welter-Stahl et al. (18) and Opitz (19)
	*L. monocytogenes*	Park et al. (20), Kim et al. (21) and Opitz et al. (22)
	*E. coli*	Kim et al. (23)
	*S. flexneri*	Girardin et al. (24) and Carneiro et al. (25)
	*C. jejuni*	Al-Sayeqh et al. (26) and Zilbauer et al. (27)
	*T. cruzi*	Silva et al. (28)
Nod2 (NLRC2)	Muramyldipeptide (MurNAc-l-Ala-d-isoGln)	Girardin et al. (8) and Inohara et al. (5)
	M-TriLys (Mur-NAc-l-Ala-d-Glu-Lys)	Girardin et al. (4)
	Respiratory syncytial virus (RSV)	Sabbah et al. (29)
	*Bacillus* species	Hasegawa et al. (13)
	*Lactobacillus* species	Hasegawa et al. (13)
	*Corynebacterium xerosis*	Hasegawa et al. (13)
	*E. coli*	Hasegawa et al. (13)
	*Pseudomonas* species	Hasegawa et al. (13)
	*M. tuberculosis*	Juárez et al. (30), Ferwerda et al. (31) and Divangahi et al. (32)
	*S. pneumoniae*	Travassos et al. (33) and Liu et al. (34)
	*C. jejuni*	Al-Sayeqh et al. (26)
	*S. flexneri*	Kufer et al. (35)
	*S. typhimurium*	Keestra et al. (36) and Hisamatsu et al. (37)
	*L. monocytogenes*	Kobayashi et al. (38)
Nalp3 (NLRP3)	Muramyldipeptide (MurNAc-l-Ala-d-isoGln)	Martinon et al. (39)
	Bacterial RNA	Kanneganti et al. (40)
	Imidazoquinoline compounds	Kanneganti et al. (40)
	MSU (monosodium urate)	Martinon et al. (41)
	CPPD (calcium pyrophosphate dihydrate)	Martinon et al. (41)
	Cholesterol crystals	Duewell et al. (42)
	Silica	Hornung et al. (43), Kuroda et al. (44) and Cassel et al. (45)
	Aluminum salts	Hornung et al. (43) and Kuroda et al. (44)
	Amyloid-beta	Halle et al. (46)
	Fatty acids	Wen et al. (47)
	Mitochondrial DNA	Nakahira et al. (48) and Shimada et al. (49)
	Aerolysin	Gurcel et al. (50)
	Maitotoxin	Mariathasan et al. (51)
	ATP	Mariathasan et al. (51)
	Nigericin	Mariathasan et al. (51)
	*S. aureus*	Craven et al. (52)
	*L. monocytogenes*	Kim et al. (53)
	*P. gingivalis*	Huang et al. (54)
	*Chlamydia* species	Abdul-Sater et al. (55) and He et al. (56)
	*Influenza A virus*	Thomas et al. (57) and Allen et al. (58)
	*Aspergillus*	Saïd-Sadier et al. (59)
	*Leishmania*	Lima-Junior et al. (60)
	ROS	Zhou et al. (61)
IPAF (NLRC4)	Cytosolic flagellin	Franchi et al. (62)
	*L. pneumophila*	Case et al. (63), Vinzing et al. (64) and Coers et al. (65)
	*S. typhimurium*	Mariathasan et al. (66), Broz et al. (67) and Miao et al. (68)
	*S. flexneri*	Suzuki et al. (69)
	*P. aeruginosa*	Cohen and Prince (70), Sutterwala et al. (71) and Franchi et al. (72)
Naip5	Cytosolic flagellin (in cooperation with IPAF)	Zamboni et al. (73)
	*L. pneumophila*	Lightfield et al. (74) and Zamboni et al. (73)

Nod1 and Nod2 have been implicated in the detection of a vast array of microbial pathogens including bacteria, parasites, and viruses. A key role for Nod1 and Nod2 in the detection of bacterial infection has been demonstrated in *Helicobacter pylori, Escherichia coli, Chlamydia* spp., *Campylobacter jejuni, Salmonella* spp., *Pseudomonas aeruginosa, S. flexneri*, and *L. monocytogenes, Mycobacterium tuberculosis*, and *Streptococcus pneumoniae* (Table 1).

More recent studies have uncovered surprising data regarding microbial recognition by Nod1 and Nod2. Nod1-deficient mice are more susceptible to infection with *Trypanosoma cruzi*, the etiological agent of Chagas disease, apparently due to the lack of a robust nitric oxide production, suggesting that Nod1 may be involved in PG-independent microbial sensing given that *T. cruzi* does not express PG (3, 28).

Supporting a role for Nod2 in the control of infections beyond bacterial/PG detection, Shaw et al. using a *Toxoplasma gondii* infection model, described a T cell intrinsic role in Nod2-deficient mice and a consequent Th1-defective immune response. In their experiments, the authors observed lower amounts of IL-2 not only during infection with *T. gondii* but also following anti-CD28 ligation. Despite the novelty of these results, T cell activation in different models appears to be normal in Nod2-deficient mice (75, 76).

Nod2 has also been implicated in the immune response to viruses. In a recent study, Sabbah et al. demonstrated that Nod2 mediated the *in vitro* production of type I IFN in cells stimulated with single stranded RNA (ssRNA) or infected with various RNA viruses. These results support the observation, made in the same study, that Nod2-deficient mice are more susceptible to respiratory syncytial virus (RSV) (29).

Finally, both Nod1 and Nod2 have been implicated in inflammatory disorders because mutations in the genes that encode these proteins were shown to be related to the establishment of genetic inflammatory diseases. The first piece of evidence of a link between mutations in *NOD2* and CD [an inflammatory bowel disease (IBD)] was provided by Hugot et al. which identified three single nucleotide polymorphisms (SNPs) in the *IBD1* locus associated with increased risk for CD (77). One of these SNPs, *Leu1007fs*, is the most common Nod2 mutation associated with the disease and encodes a protein that is no longer able to sense MDP or localize to the plasma membrane as the normal protein does upon activation (4, 78).

## Inflammasomes

By definition, inflammasomes are multimeric protein complexes that comprise a “sensor NLR” and function as platforms for the activation of pro-caspase-1, resulting in the processing of IL-1β and IL-18 and their unconventional secretion (6, 79). Several inflammasomes have been described so far, and among them, the best studied are the ones that contain NLRP3 (formerly known as NALP3) or NLRC4 (formerly known as IPAF). Many NLRs, such as NLRP1, NLRP3, and NLRC4, use the adaptor protein apoptosis-associated speck-like protein containing a CARD (ASC) to recruit pro-caspase-1, but this does not apply to all inflammasomes.

## NLRP3 Inflammasomes

NLRP3 is mostly expressed in myeloid cells and is activated by a vast array of host-derived and exogenous agonists. One common feature of NLRP3 agonists seems to be a crystalline or polymeric structure associated with danger signals or cell death. For example, monosodium urate (MSU), calcium pyrophosphate dihydrate (CPPD) (41), cholesterol crystals (42), amyloid β (80), fatty acids (47), and mtDNA (48) have all been reported to activate NLRP3. Microbial NLRP3 agonists have also been identified. NLRP3 senses bacteria, viruses, fungi, and parasites themselves or virulence factors such as pore-forming toxins. The list of pathogens detected by NLRP3 includes *Staphylococcus aureus, L. monocytogenes, Klebsiella pneumoniae, Neisseria gonorrhoeae, E. coli, Porphyromonas gingivalis, S. flexneri, Chlamydia* spp., the influenza A virus, *Aspergillus*, and more recently, *Leishmania* (Table 1).

## NLRC4 and Naip5

IPAF (also known as NLRC4) is present in the cytosol of myeloid cells, where it controls the activation of caspase-1 and IL-1β processing in response to the presence of intracellular flagellin. NLRC4 directly binds to cytosolic flagellin, an event that promotes its oligomerization through the nucleotide-binding domain (NBD) and winged-helix domain (WHD) in the presence of adenosine diphosphate (ADP) (81). The importance of IPAF-dependent activation of caspase-1 has been highlighted in infection models *in vitro* using *Salmonella typhimurium, S. flexneri, Legionella pneumophila*, and *P. aeruginosa* (66, 70, 74). In such experiments, IPAF-deficient macrophages were impaired in their ability to activate caspase-1 and secrete IL-1β and IL-18. Macrophages from IPAF-deficient mice infected with *S. typhimurium* have also been shown to be more resistant to cell death. Indeed, activation of NLRC4 leads to rapid cell death, a feature that differentiates IPAF from the other NLRs (66). Another pathogen whose detection induces cell death through IPAF is *L. pneumophila*. However, in this case, another NLR protein, Naip5 (also known as Birc1e), is required. Both Naip5 and IPAF have been reported to physically interact, but the role of Naip5 in caspase-1 activation remains to be fully elucidated, as A/J mice (mice with a mutation that results in a non-functional Naip5) are able to secrete IL-1β following infection with *S. typhimurium, P. aeruginosa*, and *L. monocytogenes* (66, 71, 73) (Table 1).

*Shigella flexneri* also triggers IPAF-dependent activation of caspase-1 and secretion of IL-1β. These data are intriguing considering that *S. flexneri* is a non-flagellated bacterium, suggesting that other factors are able to activate IPAF (69). Indeed, more recent studies revealed that *P. aeruginosa* strains lacking flagellin are still able to induce secretion of IL-1β through NLRC4 (71) (Table 1).

Although the importance of IPAF in cytosolic flagella sensing is broadly recognized, it has been demonstrated that flagellin-dependent responses may occur in the absence of IPAF. Recently, a new pathway was reported in which macrophage stimulation with flagellin leads to cell death in a cathepsin B and D-dependent manner even in IPAF-deficient cells. It has yet to be determined whether a new flagellin sensor is involved in such events (82).

## NLRX1

In contrast to the huge amount of data regarding other NLR proteins, little is known about the biological function of NLRX1. This protein is highly conserved among species and has sequence homology with Nod3. Unlike other NLRCs, NLRX1 has no CARD in its N-terminal portion but does have a putative mitochondrial-targeting sequence (83). Indeed, what we know about NLRX1 is derived from its mitochondrial localization, even though its precise localization inside this organelle is still a matter of debate. Studies from two independent groups report conflicting results; while Arnoult et al. claimed that NLRX1 is located in the mitochondrial matrix, Moore et al. proposed that the protein localizes to the outer mitochondrial membrane (84, 85). There are also discrepancies concerning the attributed function of NLRX1. Initial results from Tattoli et al. reported that NLRX1 amplifies NF-κB and JNK through the production of ROS. Opposing results from Moore et al. suggest that NLRX1 functions as a brake on innate immune pathways by inhibiting mitochondrial antiviral signaling (MAVS)-dependent NF-κB and IFN-β production upon poly I:C stimulation *in vitro* (85, 86). Further studies are required to clarify of the function of NLRX1.

## NLRP4

Very little is known about NLRP4, a 113-kDa protein also known as Nalp4 or PYPAF4. This protein is expressed in tissues as diverse as testis, oocytes, spleen, placenta, thymus, kidney, and lung. NLRP4 has been recently reported as a negative regulator of type I IFN signaling by targeting tank binding kinase-1 (TBK-1) for degradation as well as of TNF-α and IL-1β by inhibiting NF-κB activation by interacting with IKKα (87, 88).

## Autophagy

The term autophagy (meaning “self-eating”) was first introduced at the CIBA Foundation Symposium on Lysosomes in 1963 by cell biologist Christian de Duve, who also discovered lysosomes in 1955 (Nobel Prize in Physiology in 1974) and coined several other terms currently used today, such as “endocytosis” and “exocytosis.” Autophagy was first characterized by the presence of single- or double-membrane vesicles harboring cytoplasmic content in different stages of degradation – the autophagosomes. At that time, de Duve and others considered autophagy to be a non-selective degradation pathway. However, under specific circumstances, autophagy is highly specific and plays essential roles in maintaining homeostasis. For a complete historical perspective on autophagy and its importance in different pathologies, please refer Ref. (89–91).

Autophagy is a highly conserved cellular homeostatic process in which long-lived proteins, damaged organelles, or parts of the cytosol are delivered to lysosomes for degradation and recycling of functional blocks for anabolic reactions, especially during nutrient shortages. Indeed, for years, autophagy was considered a mere response to nutritional stress given that initial observations demonstrated that glucagon or amino acid deprivation triggered the formation of autophagosomes while exogenous amino acids supplementation inhibited autophagy and protein breakdown (92).

## The Machinery of Autophagosome Biogenesis

**Figure 1:**
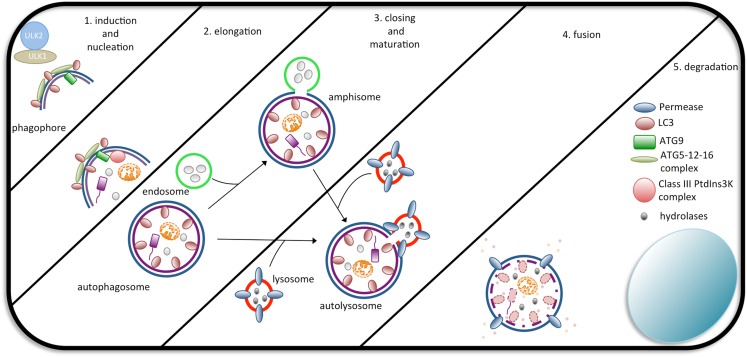
**The steps of autophagosome formation**. Macroautophagy begins with the formation of a cup-shaped structure called a phagophore as a consequence of the activity of the ULK1/2 complex. In the sequence, the ATG5-ATG12-ATG16L1 complex, Class III PtdIns3K, ATG9, and LC3 assist in the formation of the autophagosome. Autophagosomes completely wrap their cargo and fuse with endosomes (forming an amphisome, which will later fuse with lysosomes) or directly with lysosomes, forming an autolysosome. Upon fusion, the cargo is degraded by lysosomal hydrolases and exported back to the cytosol to be used by the cell.

So far, three types of autophagy have been described, chaperone-mediated autophagy (CMA), microautophagy, and macroautophagy (hereafter, autophagy) (93). The hallmark of autophagy is the generation of autophagosomes. This process occurs in a stepwise manner controlled by over 30 *Atg* genes that were initially identified in yeast species. Interestingly, most of these genes have mammalian orthologs or paralogs with high structural and functional similarities. Briefly, the process starts with the formation of a cup-shaped membrane or phagophore. Once formed, the membrane elongates and selectively and/or non-selectively enwraps the cargo (i.e., the cytosolic target), eventually sealing, completing the formation of the autophagosome. The outer membrane of the autophagosome the fuses with a lysosome membrane, forming the autolysosome, where all the degradation steps of the autophagic response take place (Figure 1). The source of the autophagosomal membrane is still a matter of debate. Various studies have proposed the plasma membrane, the endoplasmic reticulum or the outer mitochondrial membrane as the source (92).

## The Core Autophagy Pathway

At the molecular level, the number of proteins implicated in the control of autophagy is still expanding and linking autophagy with several other pathways. Here, we focus on the core of the autophagic pathway and its links to NLR signaling. For autophagosomes formation, the following two ubiquitin-like (UBL) systems are required: (i) in the Atg12 conjugation system, Atg5 and Atg12 form complexes through the covalent binding of Atg12 to the C-terminal glycine of ATG5 in UBL reactions involving Atg7 and Atg10. Atg16L1, a scaffold protein, is then conjugated to the Atg5-Atg12 complex by binding to the N-terminus of Atg5. The Atg5-Atg12-Atg16L1 complex multimerizes, forming large 800 kDa complexes that are found in the cytosol and in the forming membrane. It has been shown that the Atg16L1 complex acts as a E3-like enzyme, targeting microtubule-associated protein 1 light chain 3 (LC3) to its membrane site of lipid conjugation. (ii) The Atg8 conjugation system is crucial for inducing modifications in LC3. Under normal conditions, LC3 has a diffuse cytosolic distribution pattern. LC3 is cleaved at its C-terminus by Atg4, a cysteine protease, and undergoes UBL modifications by the E1-like enzyme, Atg7, and the E2-like enzyme, Atg3, to form LC3-I (94). During the induction of autophagy, the C-terminal carboxyl group of LC3-I is eventually conjugated with phosphatidylethanolamine to form LC3-II, which is found exclusively on the autophagosomal membrane. For this reason, LC3-II is widely used as an autophagy marker (95).

The Atg proteins, the autophagic machinery, seem to require other proteins to form autophagosomes. Similar to the Atg1-Atg13-Atg17 complex found in yeast, the Unc-51-like kinase (ULK1), focal adhesion kinase family integrating protein (FIP2000), and Atg13 proteins were described to colocalize at the nascent isolation membrane after induction of autophagy in mammalian cells (96, 97). Another protein that was shown to participate in autophagy is Atg9. Studies in mammals have demonstrated that Atg9 is essential for autophagosome formation as it associates with the trans-Golgi, endosomes, LC3, and Rab GTPases (Rab7 and Rab9) and redistributes following the induction of autophagy (92). In Figure 1, we summarize the main proteins and all the steps that are part of the formation of autophagosomes.

Interestingly, it has been proposed that autophagy may occur even in the absence of ATG proteins in both insects and mammals. However, the conditions that trigger ATG-independent autophagy and whether this type of autophagy is particular to some cell types remain to be determined (98, 99).

## Control of Autophagy

The vast number of pathways that connect to autophagy gives rise to an intricate network that makes our understanding of autophagy regulation far from complete. A major breakthrough in understanding autophagy regulation was made when the target of rapamycin (TOR) in yeast and mammals was discovered, implicating the involvement of phosphatidylinositol kinase-related kinases in the process (100–102). Both pathways are linked by the key serine/threonine kinase Akt and are known to participate in several cellular responses such as proliferation and metabolic adaptation (92). Activators of phosphatidylinositol-3 kinase (PI3K) range from cytokines to TLR ligands. After receptor activation, phosphatidylinositol-4,5-biphosphate is phosphorylated by class-I PI3K, activating Akt. mTOR complexes are effectors downstream of Akt and integrate a myriad of cellular signals, especially those related to protein synthesis and translation (103–106). Under optimal nutrient conditions, autophagy is negatively regulated by Akt and mTOR (107). This negative regulation of autophagy by mTOR has been recently shown to require inhibition of ULK kinase-complex activity through its phosphorylation (108). At least in yeast, ULK1 seems to regulate autophagy not only by inhibiting mTOR but also by interacting with Atg8 (109). It remains to be tested whether these findings also apply in mammals. Another mechanism involved in the control of mTOR activation is the recruitment of TNF receptor-associated factor 6 (TRAF6) to the lysosome by p62, where it drives the polyubiquitination of mTOR during optimal nutrient conditions (110).

Upon the induction of autophagy, vacuolar protein sorting 34 (Vps34), a class III phosphatidylinositol-3-phosphate (PtdIns3P) kinase (PI3K) enzyme, specifically phosphorylates phosphatidylinositol and is implicated in trafficking, nutrient sensing, and autophagy (111, 112). In yeast, the role of PtdIns3P seems to go beyond autophagosomal membrane elongation. It has been proposed that the levels of PtdIns3P in the phagophore assembly site (PAS) regulate autophagosome turnover due to the accumulation of ATG proteins in the membrane (113). Other important players in autophagosome formation are Vps15, beclin-1, ultraviolet radiation resistance associated gene (UVRAG), and ambra1, which together form a multiprotein complex with Vps34 that is necessary for the initial steps in autophagosome formation. For a complete view of the autophagy pathway, see the review by Boya et al. (114).

## Autophagy and Immunity to Infections

The contamination of the cytosolic compartment with invasive pathogens is a major step in the activation of innate immune defenses. In this regard, autophagy has emerged in recent years as another component of the innate immune system’s arsenal. Several microbial agents are recognized by the autophagic machinery, and their fates can vary from destruction to the creation of a replicative niche. It has been shown that the autophagic machinery can selectively segregate microbes in the cytosol. The mechanisms by which such specificity is achieved are not completely understood but seem to involve both microbial and host factors. Similar to MAMPs, which themselves were shown to induce autophagosome formation, toxins secreted by pathogens also induce autophagy (115). In a landmark paper, Nakagawa et al. demonstrated that group A *Streptococcus* (GAS) lacking streptolysin O (SLO) do not escape from phagocytic vacuoles, and thus, do not activate autophagic sequestration (116).

Gram-negative bacteria are recognized by the autophagic machinery as well. For example, *S. flexneri* uses a type 3 secretion system (T3SS) to deliver effector proteins directly into the host cell. Ogawa et al. showed that, in epithelial cells, the wild-type (WT) *S. flexneri* strain is capable of evading autophagic sequestration. This is dependent on the T3SS effector IcsB, as the mutant strain lacking IcsB is trapped by autophagy. The role of IcsB seems to be to camouflage the bacterial target molecule (VirG) from the autophagy machinery (117, 118). Interestingly, these observations seem to vary depending on cell type, given that in bone marrow-derived macrophages, no difference in bacteria sequestration was observed between the WT and IcsB mutant strains (69). The autophagic machinery apparently relies on redundant strategies to fight *Shigella*; different mechanisms have been described for the induction autophagy by this pathogen. In one mechanism, the phagocytic vacuolar membrane remnants, rather than bacterium itself, trigger autophagy in response to bacterial invasion (119).

Avoidance of autophagic destruction has also been reported for other bacteria. The *Burkholderia pseudomallei* T3SS effector, BopA, which shares some homology with IcsB, contributes to bacterial evasion from autophagosome targeting (120). The Gram-positive bacterium *L. monocytogenes* is able to invade host cells, where it finds a replicative niche within the cytosol following autophagosome escape. These events are dependent on the expression of listeriolysin O (LLO), ActA, and phospholipase C (121).

However, autophagy can serve as a back-up control mechanism for bacteria that are able to escape from other defense mechanisms. For example, after invading the host cell, *S. typhimurium* resides within vacuoles called *Salmonella*-containing vacuoles (SCV). Following SCV damage mediated by its T3SS, *S. typhimurium* gains access to the cytosol where autophagy is immediately activated to confine the bacteria and restrict the infection (122). In the case of *M. tuberculosis*, which is known to subvert host cell phagosomal maturation and survive within macrophages, autophagy induction via rapamycin or IFN-γ circumvents the phagosomal maturation blockade, leading to *M. tuberculosis* elimination (123).

Viral pathogens interact with the autophagic pathway as well. Herpes simplex virus (HSV) ICP34.5 interacts with beclin-1 and blocks autophagosome formation (124). In vesicular stomatitis virus (VSV)-infected DCs, autophagy is essential for the delivery of viral ligands to endosomes to induce type I IFN production (125).

## NLR-Mediated Autophagy and Infection

As mentioned before, the first line of host defense against infection relies on various families of PRMs. As it became evident that autophagy is also an innate immune effector mechanism, considerable efforts were made to understand the role of PMRs in the autophagic response to pathogens.

The first study linking MAMP sensing and autophagy induction was the work by Xu et al. who showed a role for TLRs in autophagy. TLRs are transmembrane proteins that recruit myeloid differentiation primary response protein 88 (MyD88) and Toll-IL-1 receptor (TIR) domain-containing adapter-inducing IFN (Trif) adapter proteins through their TIR domain to initiate downstream signaling. It was demonstrated that LPS induces autophagy in a TLR4-p38-RIP1-Trif-dependent manner (126). Later, a report from Delgado et al. showed that TLR7 could elicit similar responses upon stimulation of macrophages with ssRNA, and this was also dependent on the recruitment of MyD88 (127). The balance between Beclin-1 and Bcl-2 is a major checkpoint in the pathway for autophagy induction. Shi et al. (128) proposed that MyD88 and Trif both target Beclin-1, resulting in decreased binding to Bcl-2 and subsequent autophagy activation upon TLR stimulation. MyD88 and interferon-regulatory factors (IRFs) 5 and 7 are also recruited by mTOR to control cytokine production (128, 129).

Despite increasing evidence showing a role for TLRs in the induction of autophagy, it remains unclear how the autophagic machinery is directed to trap an entire microorganism during infection, especially considering that they are transmembrane proteins. Several bacteria, such as *Salmonella, Mycobacterium*, and *Listeria*, grow within host cells and by doing so can avoid antibody and cellular dependent defenses. Intracellular PRMs, such as the NLR family, are known for their essential role as cytosolic sentinels that can trigger robust cytokine production and inflammation. However, little was known regarding how these sensors contribute to the elimination of intracellular invaders. The first evidence implicating NLRs in autophagy-dependent control of an intracellular infection came from studies using *Drosophila* as a model. Yano et al. (130) reported that, upon infection of hemocytes with *L. monocytogenes, Drosophila* PGRP-LE detects diaminopimelic (DAP)-containing PG to trigger autophagy directed against the bacterium. Consistent with these observations, PGRP-LE null mutants were more susceptible to infection (130). The recognition of intracellular PG and subsequent induction of autophagy seem to be conserved features of the innate immune system. In 2010, two independent studies reported Nod1- and Nod2-dependent autophagy upon PG detection. We showed that Nod1 and Nod2 direct autophagy by recruiting ATG16L1 to the plasma membrane during bacterial entry into the host cell (Figure 1). Interestingly, the most common mutation in Nod2 associated with CD, Nod21007fs, results in a protein that fails to recruit ATG16L1 to initiate the formation of autophagosomes, although they still interact in the cytosol. In another study, Cooney et al. (131) found that Nod2 induces autophagy in human DCs, increasing bacterial killing, and antigen presentation. DCs expressing CD-associated variants displayed lower autophagy and antigen presentation levels upon MDP stimulation. Interestingly, while we demonstrated that the adaptor protein Rip2 and NF-κB activation is dispensable for autophagy induction because Rip2-deficient fibroblasts displayed similar numbers of *S. flexneri* targeted to autophagosomes, Cooney et al. found that Rip2-deficient DCs had reduced levels of autophagy. The difference in the cell types used could account for such differences (131, 132).

These studies gained additional relevance as a recent link between polymorphisms in ATG16L1 and CD was uncovered. CD and ulcerative colitis (UC) are common presentations of idiopathic IBD. It is estimated that their prevalence in Caucasian individuals reaches 100–150 per 100,000 (133). IBD is the outcome of combined genetic and non-genetic risk factors, and recently genome wide association studies (GWAS) have identified a non-synonymous single nucleotide polymorphism in ATG16L1 (T300A) as one of the most important genetic risk factors for CD. Studies in which the implications of this polymorphism were analyzed show that MDP-induced but not canonical autophagy is impaired in cells of individuals carrying the T300A variant (131, 132). The effect of this polymorphism on the restriction of bacterial growth varies depending on the cellular and bacterial models used. Epithelial cells expressing the T300A variant show decreased bacteria-targeted autophagy during infection with *S. typhimurium* (134). Monocytes from patients with CD and carrying the T300A allele infected with *M. avium paratuberculosis* display no difference in bacterial growth in comparison to patients with the normal allele (135). The impact of this variant on cytokine release has also been evaluated, and again, contradictory findings were observed. Plantinga et al. (136) reported that upon Nod2 stimulation with MDP (but not with TLR ligands), PBMCs from healthy volunteers carrying the T300A variant secreted increased amounts of IL-1β (136). In contrast, in another study, the same group demonstrated that PBMCs with the variant allele do not produce more IL-1β in comparison to the normal allele upon infection with *M. tuberculosis* (137).

*Legionella pneumophila*, a Gram-negative pathogen of amebae, is also able to replicate within alveolar macrophages and cause the pneumonia known as Legionnaire’s disease. Mouse macrophages, in contrast to human cells, restrict *L. pneumophila* replication through the activation of Naip5 and NLRC4 by cytosolic flagellin and activation of caspase-1 resulting in pyroptosis. In a recent study, Byrne et al. demonstrated that flagellin recognition by Naip5 and NLRC4 increases autophagosome turnover (138).

**Figure 2:**
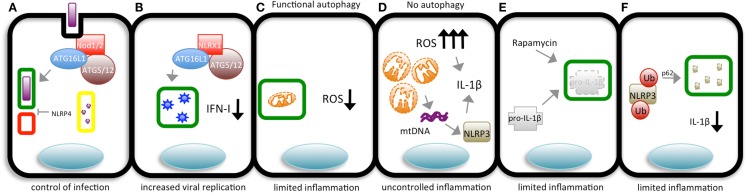
**The different means by which pathogens and autophagy interact and their outcomes**. **(A)** During bacterial invasion, Nod1/2 recruit ATG16L1 to the bacterial entry site to promote the initiation of autophagosome (green rectangle) formation, targeting the bacterium. After enclosing the pathogen, the autophagosome fuses with lysosomes (red square), giving rise to an autolysosome (yellow rectangle) in which the content is degraded by lysosomal hydrolases to control bacterial replication in the cytosol. NLRP4 can interfere in autolysosome formation. **(B)** In cells with functional autophagic machinery, damaged mitochondria are targeted to autophagosomes (green rectangles) avoiding the accumulation of ROS and limiting inflammation. **(C)** Cells in which autophagy is impaired accumulate swollen mitochondria and high levels of ROS, leading to uncontrolled IL-1β production. Alternatively, damaged mitochondria release mitochondrial DNA (mtDNA), which is sensed by NLRP3 that, together with ROS, induces the production of IL-1β. **(D)** Inflammasome sensors are ubiquitinated and targeted to autophagosomes (green rectangles) for degradation, limiting the production of IL-1β. **(E)** Pro-IL-1β is targeted to autophagosomes (green rectangles) upon autophagy induction (by rapamycin) to limit the magnitude of inflammation. **(F)** NLRX1 associates with ATG16L1 and the ATG5/12 complex to induce the formation of autophagosomes (green rectangles) targeting VSV, which later fuse with lysosomes (red square), forming autolysosomes (yellow rectangles) in which the virus is degraded.

NLRX1 has been shown to enhance autophagy. A recent study demonstrated that NLRX1 enhances autophagy through interaction with the Tu translation elongation factor (TUFM) which, in turn, interacts with the Atg5-Atg12 complex. It is not clear, however, how this NLRX1-TUFM-Atg5-Atg12 interaction leads to increased autophagy. Still, NLRX1 plays an important role as a pro-autophagic factor during vesicular stomatitis (VSV) infection. Lei et al. (139) showed decreased viral replication in NLRX1-deficient fibroblasts, suggesting that autophagy is important for VSV replication, although a previous work demonstrated that VSV succumbs to autophagy in a *Drosophila* model (139, 140). Of note is the fact that the role of NLRX1 in autophagy varies among studies using the different NLRX1 knockout mice available. While Soares et al. and Rebsamen et al. found that the MAVS pathway is fully functional in their NLRX1-deficient mice, Allen et al. reported an enhancement in this signaling pathway using a different NLRX1 knockout mice [the same used by Lei et al. (139, 141–143)]. It remains to be determined whether the role of NLRX1 in autophagy is specific to the knockout animals used by Lei et al. or if it is a general feature of all NLRX1-deficient mice (Figure 2B).

Finally, in contrast to what was described above, NLRs can also act as negative regulators of autophagy through mechanisms that are not yet completely elucidated. Suzuki et al. reported that NLRC4- and caspase-1-deficient macrophages display increased targeting of *S. flexneri* to autophagosomes (69). Similar results were found in a study analyzing the impact of NLRC4 and NLRP4 on autophagy, where it was demonstrated that epithelial cells with both proteins silenced displayed enhanced autophagy. A partial explanation for these observations is that NLRP4 is part of the Beclin-1 and C-VPS (a complex consisting of VPS11, VPS16, VPS18, and Rab7 that controls membrane tethering and fusion of vacuolar membranes) complexes, which are essential for the biogenesis and maturation of autophagosomes, respectively (144). The elucidation of the precise mechanisms by which NLRP4 exerts its negative effects on autophagy requires further studies (Figure 2A).

## Autophagy-Dependent Control of NLR-Dependent Inflammation

Another function of autophagy seems to be the control of the magnitude of inflammatory responses (145). In the last few years, several groups have reported that autophagy blockade by pharmacological or genetic means leads to increased production of cytokines by different mechanisms. Two independent studies have shown that ATG5-deficient fibroblasts produce significantly more IFN-I and IL-6 then WT cells during viral infection. The mechanisms likely involve the interaction between ATG12-ATG5 complexes with IPS-1, RIG-I, and MDA-5 and accumulation of damaged mitochondria in the autophagy-deficient cells, as discussed below (146, 147).

## Autophagy and Inflammasomes

### A role for ROS

As discussed above, the cells deficient in ATG genes produce increased levels of pro-inflammatory cytokines (146, 147). Tal et al. (147) demonstrated a vital role of autophagy in the removal of damaged mitochondria (mitophagy), and thus, in cell homeostasis. Cells with defects in autophagy, such as Atg5-deficient cells, accumulate damaged mitochondria, and consequently present increased levels of ROS. This in turn results in the enhancement of the levels of IFNα and IL-6 production upon infection with VSV. In addition, the authors show that by using the antioxidants *N*-acetyl-*L*-cysteine (NAC) and propyl-gallate (PG) during VSV infection, they were able to revert the increase in cytokine production. The removal of damaged mitochondria can be regulated by adapter proteins that participate in NLR-activating pathways. For example, besides its well-known and crucial role linking Nod1 and Nod2 sensing to NF-κB activation, Rip2 has also been implicated in mitophagy. In a recent study, Lupfer et al. showed that Rip2 regulates mitophagy through ULK1 to keep ROS at basal levels. Genetic deletion of Rip2 leads to the accumulation of ROS and significantly higher levels of IL-18 and IFN-γ upon influenza infection (148) (Figure 2C).

Among all the cytokines that have been studied, IL-1β seems to be the cytokine whose production is most dramatically affected by autophagy. Processing and activation of pro-IL-1β into its active form depends on the assembly of inflammasomes. Increased ROS levels seem to be a prerequisite for inflammasome activation. The manipulation of autophagy by pharmacological or genetic means has a profound impact on IL-1β production and secretion during infection or LPS treatment. Indeed, the use of the autophagy inhibitor 3-methyladenine (3-MA), deletion of LC3B or ATG16L1, silencing of Beclin-1 or dominant negative forms of the cysteine protease ATG4B all lead to remarkably higher amounts of IL-1β (48, 149–151). The first evidence to show that autophagy can modulate IL-1β production surfaced in 2008 with the work of Saitoh et al. These authors reported that macrophages from ATG16L1-deficient mice produced much higher levels of IL-1β after LPS exposure and that this was not due to defects in the generation of pro-IL-1β and pro-caspase-1. These authors proposed a model in which TRIF and loss of K^+^ and ROS are required for the activation of inflammasomes, and subsequently, IL-1β processing (150). However, it remains to be shown that these observations were not due to defective mitophagy. Further studies not only confirmed but expanded the evidence for the requirement of functional autophagy for the maintenance of basal levels of ROS, and subsequently, for the control of inflammasome activation. In an elegant study, Nakahira et al. dissected the role of autophagy in the activation of the NLRP3 inflammasome. In a series of experiments in which autophagy was inhibited either by deletion of LC3B or by heterozygous deletion of Beclin-1, a robust increase in IL-1β processing and release was observed following stimulation with LPS plus ATP. According to the authors, these stimuli led to swollen mitochondria and the release of its DNA (mtDNA). In ρ^0^ J774A.1 macrophages, the release of mtDNA into the cytosol is blocked, and IL-1β secretion is impaired. Similar results were obtained when cells were treated with DNAse I. Interestingly, the production of ROS and consequent activation of the inflammasome were dependent on the presence of cytosolic mtDNA. Altogether, the results from this study delineate a model in which LPS plus ATP induce mitochondrial damage, ROS production, and NLRP3-dependent release of mtDNA into the cytosol, resulting in the activation of caspase-1 and release of IL-1β (48). The findings reported by Nakahira et al. were partially contradicted by a more recent study that suggests that ATP actually induces the release of oxidized mtDNA, which in turn binds to NLRP3 to induce IL-1β production. Oxidized mtDNA was not detected in the cytosol of NLPR3-deficient macrophages. According to the authors, this could be explained by the fact that unbound oxidized mtDNA is rapidly degraded, but this later observation still lacks experimental confirmation (49) (Figure 2D). It is important to note that the idea of ROS as an activator of NLPR3 inflammasomes was challenged by a study demonstrating that ROS is key for priming of NLRP3 but not for its activation. One way or another, it is clear that ROS is necessary for IL-1β production (152).

The source of ROS required for inflammasome activation is also a matter of debate. Initial reports suggested that nicotinamide dinucleotide phosphate (NADPH) oxidases were the main source for inflammasome activation (153). The NADPH complex comprises the membrane-bound gp91^phox^ and p22^phox^ glycoproteins and the cytosolic components p47^phox^ and p67^phox^. Patients with chronic granulomatous disease (CGD) can have mutations in any of the NADPH oxidases and as a result present defective phagocytes because their cells have impaired capacity to generate superoxide anion and its metabolites, hydrogen peroxide, the hydroxyl anion, and hypohalous acid (154). Meissner et al. reported that in monocytes from CGD patients with mutations in gp91^phox^, p22^phox^, and p47^phox^, stimulation with LPS plus ATP led to the activation of caspase-1 and secretion of mature IL-1β (155). Furthermore, monocytes from CGD patients presented elevated IL-1β levels in comparison to monocytes from healthy controls (154). These findings challenged the notion that NADPH oxidases are the source of ROS necessary to induce inflammasome activation. This question was apparently clarified by the work of Zhou et al. which demonstrated that ROS generated by dysfunctional mitochondria [mtROS, achieved by either by treating cells with the complex I inhibitor, rotenone, or silencing of the voltage-dependent anion channel (VDAC)] activates the Nlrp3 inflammasome. The requirement of mtROS for activation of the inflammasome seems to be specific for Nlrp3 because VDAC1 silencing did not influence activation of NLRC4 of AIM2 inflammasomes. Once again, the crucial role of autophagy in clearing damaged mitochondria was demonstrated in the work of Zhou et al. in which 3-MA treatment or beclin-1 or Atg5 silencing resulted in ROS accumulation and inflammasome activation (61).

As already mentioned, mutations in NLR genes are associated with increased risk for inflammatory diseases. Auto-activation of the NLRP3 inflammasome has been linked to several autosomal dominant cryopyrinopathies or cryopyrin-associated periodic fever syndromes (CAPS), such as familial cold-induced autoinflammatory syndrome (FCAS), Muckle–Wells syndrome (MWS), and neonatal onset multisystem inflammatory disorder or chronic infantile neurologic cutaneous and articular syndrome (NOMID/CINCA) (156). These syndromes, despite their different names, represent a continuum of disease severity where FCAS is the mildest and NOMID/CINCA the most severe. These cryopyrinopathies are associated with periodic fever, rashes, arthralgia, and conjunctivitis, and the aberrant production of IL-1β is the most prominent feature related to all these manifestations (6).

The increasing incidence of T2D has become a global health burden. TD2 has been associated with low-grade inflammation that leads to insulin resistance. In this context, IL-1β is one of the main cytokines implicated in T2D, mediating the destruction of beta cells and resulting in insulin resistance in cells that were initially sensitive to the hormone. A high-fat diet (HFD) is one of the factors associated with T2D. Indeed, T2D patients display augmented levels of free fatty acids in the serum. In a recent study, Wen et al. demonstrated in a bone marrow-derived model that palmitate, an abundant saturated fatty acid in the plasma, inhibits AMP-activated protein kinase (AMPK), leading to defective autophagy, and thus, ROS accumulation. These events contribute to the elevation of IL-1β production and impairment of insulin signaling *in vitro* (47).

A recent report showed that autophagy is involved not only in IL-1β production but also in its secretion. Macrophages from Atg5 conditional knockout mice secreted significantly more IL-1β during the induction of autophagy triggered by starvation. These observations need further confirmation (157).

### A role for ubiquitin

As in many aspects of its biology, autophagy has ambiguous roles in regulating inflammasome activation and acts both as a positive and negative regulator depending on the experimental model.

In a recent study, Harris et al. provided experimental evidence that autophagy controls inflammasomes by targeting its components to autophagosomes. These authors showed that pro-IL-1β is delivered to autophagosomes after TLR stimulation. Upon autophagy induction by rapamycin treatment, pro-IL-1β is degraded, limiting the amount available for the processing and secretion of IL-1β (149) (Figure 2E).

One mechanism underlying the targeting of inflammasome proteins to autophagosomes seems to be ubiquitination. In a recent study by Shi et al. it was demonstrated that inflammasomes containing ASC are directed to autophagosomes during NLRP3 or AIM2 activation in primary macrophages. They also provided evidence that beclin-1 and p62 are involved in targeting ASC to autophagosomes after it is K63 ubiquitination. The results presented suggest that by using its separate UBA and LIR domains, p62/SQTM1 bridges ASC K623 ubiquitination, and autophagy-dependent degradation (151). In addition to ASC, NLRP3 is also ubiquitinated in a mtDNA-, ROS-, and ATP-dependent manner, but its delivery into autophagosomes has not yet been demonstrated (158) (Figure 2F).

## Concluding Remarks

The NLR and autophagy fields are two exciting research areas in biology with many unanswered questions related to the precise mechanisms that coordinate the “talk” between NLR proteins and autophagy. It will be interesting to discover in more detail whether autophagy modulation can be used to control NLR-dependent immune pathways to improve therapeutic strategies for inflammatory and infectious diseases. In light of the recent findings that connect autophagy and inflammasome regulation, it remains to be determined whether alterations in autophagy could explain, at least in part, the dysregulated production of IL-1β in NLRP3-associated cryopyrinopathies. We believe that in the near future, some of the findings discussed in the present review have the potential to be translated into new therapeutic strategies that can be applied in daily medical practice.

## Conflict of Interest Statement

The authors declare that the research was conducted in the absence of any commercial or financial relationships that could be construed as a potential conflict of interest.

## References

[1] Philpott DJ Yamaoka S Israel A Sansonetti PJ Invasive *Shigella flexneri* activates NF-kappa B through a lipopolysaccharide-dependent innate intracellular response and leads to IL-8 expression in epithelial cells. J Immunol (2000) 165:903–14 10878365 10.4049/jimmunol.165.2.903

[2] Fritz JH Ferrero RL Philpott DJ Girardin SE Nod-like proteins in immunity, inflammation and disease. Nat Immunol (2006) 7:1250–7 10.1038/ni1412 17110941

[3] Bonardi V Cherkis K Nishimura MT Dangl JL A new eye on NLR proteins: focused on clarity or diffused by complexity? Curr Opin Immunol (2012) 24:41–50 10.1016/j.coi.2011.12.006 22305607 PMC3482489

[4] Girardin SE Boneca IG Viala J Chamaillard M Labigne A Thomas G Nod2 is a general sensor of peptidoglycan through muramyl dipeptide (MDP) detection. J Biol Chem (2003) 278:8869–72 10.1074/jbc.C200651200 12527755

[5] Inohara N Ogura Y Fontalba A Gutierrez O Pons F Crespo J Host recognition of bacterial muramyl dipeptide mediated through NOD2. Implications for Crohn’s disease. J Biol Chem (2003) 278:5509–12 10.1074/jbc.C200673200 12514169

[6] Carneiro L Magalhaes JG Tattoli I Philpott DJ Travassos LH Nod-like proteins in inflammation and disease. J Pathol (2008) 214:136–48 10.1002/path.2271 18161746

[7] Travassos LH Carneiro LAM Girardin SE Boneca IG Lemos R Bozza MT Nod1 participates in the innate immune response to *Pseudomonas aeruginosa*. J Biol Chem (2005) 280:36714–8 10.1074/jbc.M501649200 16150702

[8] Girardin SE Boneca IG Carneiro LAM Antignac A Jéhanno M Viala J Nod1 detects a unique muropeptide from gram-negative bacterial peptidoglycan. Science (2003) 300:1584–7 10.1126/science.1084677 12791997

[9] Chamaillard M Hashimoto M Horie Y Masumoto J Qiu S Saab L An essential role for NOD1 in host recognition of bacterial peptidoglycan containing diaminopimelic acid. Nat Immunol (2003) 4:702–7 10.1038/ni945 12796777

[10] Uehara A Yang S Fujimoto Y Fukase K Kusumoto S Shibata K Muramyldipeptide and diaminopimelic acid-containing desmuramylpeptides in combination with chemically synthesized toll-like receptor agonists synergistically induced production of interleukin-8 in a NOD2- and NOD1-dependent manner, respectively, in human monocytic cells in culture. Cell Microbiol (2005) 7:53–61 10.1111/j.1462-5822.2004.00433.x 15617523

[11] Magalhaes JG Philpott DJ Nahori M-A Jéhanno M Fritz J Bourhis LL Murine Nod1 but not its human orthologue mediates innate immune detection of tracheal cytotoxin. EMBO Rep (2005) 6:1201–7 10.1038/sj.embor.7400552 16211083 PMC1369207

[12] Uehara A Fujimoto Y Kawasaki A Kusumoto S Fukase K Takada H Meso-diaminopimelic acid and meso-lanthionine, amino acids specific to bacterial peptidoglycans, activate human epithelial cells through NOD1. J Immunol (2006) 177:1796–804 16849490 10.4049/jimmunol.177.3.1796

[13] Hasegawa M Yang K Hashimoto M Park J-H Kim Y-G Fujimoto Y Differential release and distribution of Nod1 and Nod2 immunostimulatory molecules among bacterial species and environments. J Biol Chem (2006) 281:29054–63 10.1074/jbc.M602638200 16870615

[14] Le Bourhis L Magalhaes JG Selvanantham T Travassos LH Geddes K Fritz JH Role of Nod1 in mucosal dendritic cells during *Salmonella* pathogenicity island 1-independent *Salmonella enterica serovar typhimurium* infection. Infect Immun (2009) 77:4480–6 10.1128/IAI.00519-09 19620349 PMC2747964

[15] Viala J Chaput C Boneca IG Cardona A Girardin SE Moran AP Nod1 responds to peptidoglycan delivered by the *Helicobacter pylori* cag pathogenicity island. Nat Immunol (2004) 5:1166–74 10.1038/ni1131 15489856

[16] Kavathas PB Boeras CM Mulla MJ Abrahams VM Nod1, but not the ASC inflammasome, contributes to induction of IL-1β secretion in human trophoblasts after sensing of *Chlamydia trachomatis*. Mucosal Immunol (2013) 6:235–43 10.1038/mi.2012.63 22763410 PMC3465624

[17] Buchholz KR Stephens RS The cytosolic pattern recognition receptor NOD1 induces inflammatory interleukin-8 during *Chlamydia trachomatis* infection. Infect Immun (2008) 76:3150–5 10.1128/IAI.00104-08 18426885 PMC2446689

[18] Welter-Stahl L Ojcius DM Viala J Girardin S Liu W Delarbre C Stimulation of the cytosolic receptor for peptidoglycan, Nod1, by infection with *Chlamydia trachomatis* or *Chlamydia muridarum*. Cell Microbiol (2006) 8:1047–57 10.1111/j.1462-5822.2006.00686.x 16681844

[19] Opitz B Nod1-mediated endothelial cell activation by *Chlamydophila pneumoniae*. Circ Res (2005) 96:319–26 10.1161/01.RES.0000155721.83594.2c 15653568

[20] Park J-H Kim Y-G Shaw M Kanneganti T-D Fujimoto Y Fukase K Nod1/RICK and TLR signaling regulate chemokine and antimicrobial innate immune responses in mesothelial cells. J Immunol (2007) 179:514–21 17579072 10.4049/jimmunol.179.1.514

[21] Kim Y-G Park J-H Daignault S Fukase K Nuñez G Cross-tolerization between Nod1 and Nod2 signaling results in reduced refractoriness to bacterial infection in Nod2-deficient macrophages. J Immunol (2008) 181:4340–6 18768892 10.4049/jimmunol.181.6.4340 PMC2830743

[22] Opitz B Püschel A Beermann W Hocke AC Förster S Schmeck B *Listeria monocytogenes* activated p38 MAPK and induced IL-8 secretion in a nucleotide-binding oligomerization domain 1-dependent manner in endothelial cells. J Immunol (2006) 176:484–90 16365441 10.4049/jimmunol.176.1.484

[23] Kim JG Lee SJ Kagnoff MF Nod1 is an essential signal transducer in intestinal epithelial cells infected with bacteria that avoid recognition by toll-like receptors. Infect Immun (2004) 72:1487–95 10.1128/IAI.72.3.1487-1495.2004 14977954 PMC356064

[24] Girardin SE Tournebize R Mavris M Page AL Li X CARD4/Nod1 mediates NF-κB and JNK activation by invasive *Shigella flexneri*. EMBO Rep (2001) 2(8):736–42 10.1093/embo-reports/kve155 11463746 PMC1083992

[25] Carneiro LAM Travassos LH Soares F Tattoli I Magalhaes JG Bozza MT *Shigella* induces mitochondrial dysfunction and cell death in nonmyleoid cells. Cell Host Microbe (2009) 5:123–36 10.1016/j.chom.2008.12.011 19218084

[26] Al-Sayeqh AF Loughlin MF Dillon E Mellits KH Connerton IF *Campylobacter jejuni* activates NF-kappaB independently of TLR2, TLR4, Nod1 and Nod2 receptors. Microb Pathog (2010) 49:294–304 10.1016/j.micpath.2010.06.011 20599492

[27] Zilbauer M Dorrell N Elmi A Lindley KJ Schüller S Jones HE A major role for intestinal epithelial nucleotide oligomerization domain 1 (NOD1) in eliciting host bactericidal immune responses to *Campylobacter jejuni*. Cell Microbiol (2007) 9:2404–16 10.1111/j.1462-5822.2007.01008.x 17521327

[28] Silva GK Gutierrez FRS Guedes PMM Horta CV Cunha LD Mineo TWP Cutting edge: nucleotide-binding oligomerization domain 1-dependent responses account for murine resistance against *Trypanosoma cruzi* infection. J Immunol (2010) 184:1148–52 10.4049/jimmunol.0902254 20042586

[29] Sabbah A Chang TH Harnack R Frohlich V Tominaga K Dube PH Activation of innate immune antiviral responses by Nod2. Nat Immunol (2009) 10:1073–80 10.1038/ni.1782 19701189 PMC2752345

[30] Juárez E Carranza C Hernández-Sánchez F León-Contreras JC Hernández-Pando R Escobedo D NOD2 enhances the innate response of alveolar macrophages to *Mycobacterium tuberculosis* in humans. Eur J Immunol (2012) 42:880–9 10.1002/eji.201142105 22531915

[31] Ferwerda G Girardin SE Kullberg B-J Le Bourhis L de Jong DJ Langenberg DML NOD2 and toll-like receptors are nonredundant recognition systems of *Mycobacterium tuberculosis*. PLoS Pathog (2005) 1:e34 10.1371/journal.ppat.0010034 PMC1291354 16322770

[32] Divangahi M Mostowy S Coulombe F Kozak R Guillot L Veyrier F NOD2-deficient mice have impaired resistance to *Mycobacterium tuberculosis* infection through defective innate and adaptive immunity. J Immunol (2008) 181:7157–65 18981137 10.4049/jimmunol.181.10.7157

[33] Travassos LH Girardin SE Philpott DJ Blanot D Nahori M-A Werts C Toll-like receptor 2-dependent bacterial sensing does not occur via peptidoglycan recognition. EMBO Rep (2004) 5:1000–6 10.1038/sj.embor.7400248 15359270 PMC1299148

[34] Liu X Chauhan VS Young AB Marriott I NOD2 mediates inflammatory responses of primary murine glia to *Streptococcus pneumoniae*. Glia (2010) 58:839–47 10.1002/glia.20968 20091781 PMC2967038

[35] Kufer TA Kremmer E Banks DJ Philpott DJ Role for erbin in bacterial activation of Nod2. Infect Immun (2006) 74:3115–24 10.1128/IAI.00035-06 16714539 PMC1479233

[36] Keestra AM Winter MG Klein-Douwel D Xavier MN Winter SE Kim A A *Salmonella* virulence factor activates the NOD1/NOD2 signaling pathway. MBio (2011) 2(6):e00266–11 10.1128/mBio.00266-11 22186610 PMC3238304

[37] Hisamatsu T Suzuki M Reinecker H-C Nadeau WJ McCormick BA Podolsky DK CARD15/NOD2 functions as an antibacterial factor in human intestinal epithelial cells. Gastroenterology (2003) 124:993–1000 10.1053/gast.2003.50153 12671896

[38] Kobayashi M Wilson AC Chao MV Mohr I Control of viral latency in neurons by axonal mTOR signaling and the 4E-BP translation repressor. Genes Dev (2012) 26:1527–32 10.1101/gad.190157.112 22802527 PMC3404381

[39] Martinon F Agostini L Meylan E Tschopp J Identification of bacterial muramyl dipeptide as activator of the NALP3/cryopyrin inflammasome. Curr Biol (2004) 14(21):1929–34 10.1016/j.cub.2004.10.027 15530394

[40] Kanneganti TD Ozoren N Body-Malapel M Amer A Bacterial RNA and small antiviral compounds activate caspase-1 through cryopyrin/Nalp3. Nature (2006) 440(7081):233–6 10.1038/nature04517 16407888

[41] Martinon F Pétrilli V Mayor A Tardivel A Tschopp J Gout-associated uric acid crystals activate the NALP3 inflammasome. Nat Cell Biol (2006) 440:237–41 10.1038/nature04516 16407889

[42] Duewell P Kono H Rayner KJ Sirois CM Vladimer G Bauernfeind FG NLRP3 inflammasomes are required for atherogenesis and activated by cholesterol crystals. Nature (2010) 464:1357–61 10.1038/nature08938 20428172 PMC2946640

[43] Hornung V Bauernfeind F Halle A Samstad EO Kono H Rock KL Silica crystals and aluminum salts activate the NALP3 inflammasome through phagosomal destabilization. Nat Immunol (2008) 9:847–56 10.1038/ni.1631 18604214 PMC2834784

[44] Kuroda E Ishii KJ Uematsu S Ohata K Coban C Akira S Silica crystals and aluminum salts regulate the production of prostaglandin in macrophages via NALP3 inflammasome-independent mechanisms. Immunity (2011) 34:514–26 10.1016/j.immuni.2011.03.019 21497116

[45] Cassel SL Eisenbarth SC Iyer SS Sadler JJ Colegio OR Tephly LA The Nalp3 inflammasome is essential for the development of silicosis. Proc Natl Acad Sci U S A (2008) 105:9035–40 10.1073/pnas.0803933105 18577586 PMC2449360

[46] Halle A Hornung V Petzold GC Stewart CR Monks BG Reinheckel T The NALP3 inflammasome is involved in the innate immune response to amyloid-beta. Nat Immunol (2008) 9:857–65 10.1038/ni.1636 18604209 PMC3101478

[47] Wen H Gris D Lei Y Jha S Zhang L Huang MT-H Fatty acid-induced NLRP3-ASC inflammasome activation interferes with insulin signaling. Nat Immunol (2011) 12:408–15 10.1038/ni.2022 21478880 PMC4090391

[48] Nakahira K Haspel JA Rathinam VAK Lee S-J Dolinay T Lam HC Autophagy proteins regulate innate immune responses by inhibiting the release of mitochondrial DNA mediated by the NALP3 inflammasome. Nat Immunol (2011) 12:222–30 10.1038/ni.1980 21151103 PMC3079381

[49] Shimada K Crother TR Karlin J Dagvadorj J Chiba N Chen S Oxidized mitochondrial DNA activates the NLRP3 inflammasome during apoptosis. Immunity (2012) 36:401–14 10.1016/j.immuni.2012.01.009 22342844 PMC3312986

[50] Gurcel L Abrami L Girardin S Tschopp J van der Goot FG Caspase-1 activation of lipid metabolic pathways in response to bacterial pore-forming toxins promotes cell survival. Cell (2006) 126:1135–45 10.1016/j.cell.2006.07.033 16990137

[51] Mariathasan S Weiss DS Newton K McBride J O’Rourke K Roose-Girma M Cryopyrin activates the inflammasome in response to toxins and ATP. Nature (2006) 440:228–32 10.1038/nature04515 16407890

[52] Craven RR Gao X Allen IC Gris D Wardenburg JB *Staphylococcus aureus* α-hemolysin activates the NLRP3-inflammasome in human and mouse monocytic cells. PLoS One (2009) 4(10):e7446 10.1371/journal.pone.0007446 19826485 PMC2758589

[53] Kim S Bauernfeind F Ablasser A *Listeria monocytogenes* is sensed by the NLRP3 and AIM2 inflammasome. Eur J Immunol (2010) 40:1545–51 10.1002/eji.201040425 20333626 PMC3128919

[54] Huang MT-H Taxman DJ Holley-Guthrie EA Moore CB Willingham SB Madden V Critical role of apoptotic speck protein containing a caspase recruitment domain (ASC) and NLRP3 in causing necrosis and ASC speck formation induced by *Porphyromonas gingivalis* in human cells. J Immunol (2009) 182:2395–404 10.4049/jimmunol.0800909 19201894

[55] Abdul-Sater AA Koo E Häcker G Ojcius DM Inflammasome-dependent caspase-1 activation in cervical epithelial cells stimulates growth of the intracellular pathogen *Chlamydia trachomatis*. J Biol Chem (2009) 284:26789–96 10.1074/jbc.M109.026823 19648107 PMC2785367

[56] He X Mekasha S Mavrogiorgos N Inflammation and fibrosis during *Chlamydia pneumoniae* infection is regulated by IL-1 and the NLRP3/ASC inflammasome. J Immunol (2010) 184:5743–54 10.4049/jimmunol.0903937 20393140 PMC3156096

[57] Thomas PG Dash P Aldridge JR Jr Ellebedy AH The intracellular sensor NLRP3 mediates key innate and healing responses to influenza A virus via the regulation of caspase-1. Immunity (2009) 30(4):566–75 10.1016/j.immuni.2009.02.006 19362023 PMC2765464

[58] Allen IC Scull MA Moore CB Holl EK The NLRP3 inflammasome mediates in vivo innate immunity to influenza A virus through recognition of viral RNA. Immunity (2009) 30(4):556–65 10.1016/j.immuni.2009.02.005 19362020 PMC2803103

[59] Saïd-Sadier N Padilla E Langsley G Ojcius DM *Aspergillus fumigatus* stimulates the NLRP3 inflammasome through a pathway requiring ROS production and the Syk tyrosine kinase. PLoS One (2010) 5:e10008 10.1371/journal.pone.0010008 20368800 PMC2848854

[60] Lima-Junior DS Costa DL Carregaro V Cunha LD Silva ALN Mineo TWP Inflammasome-derived IL-1β production induces nitric oxide-mediated resistance to *Leishmania*. Nat Med (2013) 19:909–15 10.1038/nm.3221 23749230

[61] Zhou R Yazdi AS Menu P Tschopp J A role for mitochondria in NLRP3 inflammasome activation. Nature (2010) 469:221–5 10.1038/nature09663 21124315

[62] Franchi L Amer A Body-Malapel M Kanneganti T-D Özören N Jagirdar R Cytosolic flagellin requires Ipaf for activation of caspase-1 and interleukin 1β in *Salmonella*-infected macrophages. Nat Immunol (2006) 7:576–82 10.1038/ni1346 16648852

[63] Case CL Shin S Roy CR Asc and Ipaf inflammasomes direct distinct pathways for caspase-1 activation in response to *Legionella pneumophila*. Infect Immun (2009) 77:1981–91 10.1128/IAI.01382-08 19237518 PMC2681768

[64] Vinzing M Eitel J Lippmann J Hocke AC Zahlten J Slevogt H NAIP and Ipaf control *Legionella pneumophila* replication in human cells. J Immunol (2008) 180:6808–15 18453601 10.4049/jimmunol.180.10.6808

[65] Coers J Vance RE Fontana MF Dietrich WF Restriction of *Legionella pneumophila* growth in macrophages requires the concerted action of cytokine and Naip5/Ipaf signalling pathways. Cell Microbiol (2007) 9:2344–57 10.1111/j.1462-5822.2007.00963.x 17506816

[66] Mariathasan S Newton K Monack DM Vucic D French DM Lee WP Differential activation of the inflammasome by caspase-1 adaptors ASC and Ipaf. Nature (2004) 430:213–8 10.1038/nature02664 15190255

[67] Broz P Newton K Lamkanfi M Mariathasan S Dixit VM Monack DM Redundant roles for inflammasome receptors NLRP3 and NLRC4 in host defense against *Salmonella*. J Exp Med (2010) 207:1745–55 10.1084/jem.20100257 20603313 PMC2916133

[68] Miao EA Mao DP Yudkovsky N Bonneau R Lorang CG Warren SE From the cover: innate immune detection of the type III secretion apparatus through the NLRC4 inflammasome. Proc Natl Acad Sci U S A (2010) 107:3076–80 10.1073/pnas.0913087107 20133635 PMC2840275

[69] Suzuki T Franchi L Toma C Ashida H Ogawa M Yoshikawa Y Differential regulation of caspase-1 activation, pyroptosis, and autophagy via Ipaf and ASC in *Shigella*-infected macrophages. PLoS Pathog (2007) 3:e111 10.1371/journal.ppat.0030111 17696608 PMC1941748

[70] Cohen TS Prince AS Activation of inflammasome signaling mediates pathology of acute *P. aeruginosa* pneumonia. J Clin Invest (2013) 123:1630–7 10.1172/JCI66142 23478406 PMC3613922

[71] Sutterwala FS Mijares LA Li L Ogura Y Kazmierczak BI Flavell RA Immune recognition of *Pseudomonas aeruginosa* mediated by the IPAF/NLRC4 inflammasome. J Exp Med (2007) 204:3235–45 10.1084/jem.20071239 18070936 PMC2150987

[72] Franchi L Stoolman J Kanneganti T-D Verma A Ramphal R Nuñez G Critical role for Ipaf in *Pseudomonas aeruginosa*-induced caspase-1 activation. Eur J Immunol (2007) 37:3030–9 10.1002/eji.200737532 17935074

[73] Zamboni DS Kobayashi KS Kohlsdorf T Ogura Y Long EM Vance RE The Birc1e cytosolic pattern-recognition receptor contributes to the detection and control of *Legionella pneumophila* infection. Nat Immunol (2006) 7:318–25 10.1038/ni1305 16444259

[74] Lightfield KL Persson J Trinidad NJ Brubaker SW Kofoed EM Sauer J-D Differential requirements for NAIP5 in activation of the NLRC4 inflammasome. Infect Immun (2011) 79:1606–14 10.1128/IAI.01187-10 21282416 PMC3067536

[75] Shaw MH Reimer T Sánchez-Valdepeñas C Warner N Kim Y-G Fresno M T cell-intrinsic role of Nod2 in promoting type 1 immunity to *Toxoplasma gondii*. Nat Immunol (2009) 10:1267–74 10.1038/ni.1816 19881508 PMC2803073

[76] Caetano BC Biswas A Lima DS Benevides L Mineo TWP Horta CV Intrinsic expression of Nod2 in CD4+ T lymphocytes is not necessary for the development of cell-mediated immunity and host resistance to *Toxoplasma gondii*. Eur J Immunol (2011) 41:3627–31 10.1002/eji.201141876 22002196 PMC3241608

[77] Hugot JP Chamaillard M Zouali H Lesage S Cezard JP Belaiche J Association of NOD2 leucine-rich repeat variants with susceptibility to Crohn’s disease. Nature (2001) 411:599–603 10.1038/35079107 11385576

[78] Barnich N Aguirre JE Reinecker H-C Xavier R Podolsky DK Membrane recruitment of NOD2 in intestinal epithelial cells is essential for nuclear factor-{kappa}B activation in muramyl dipeptide recognition. J Cell Biol (2005) 170:21–6 10.1083/jcb.200502153 15998797 PMC2171381

[79] Latz E Xiao TS Stutz A Activation and regulation of the inflammasomes. Nat Rev Immunol (2013) 13:397–411 10.1038/nri3452 23702978 PMC3807999

[80] Masters SL Dunne A Subramanian SL Hull RL Tannahill GM Sharp FA Activation of the NLRP3 inflammasome by islet amyloid polypeptide provides a mechanism for enhanced IL-1β in type 2 diabetes. Nat Immunol (2010) 11:897–904 10.1038/ni.1935 20835230 PMC3103663

[81] Hu Z Yan C Liu P Huang Z Ma R Zhang C Crystal structure of NLRC4 reveals its autoinhibition mechanism. Science (2013) 341:172–5 10.1126/science.1236381 23765277

[82] Lage SL Buzzo CL Amaral EP Matteucci KC Massis LM Icimoto MY Cytosolic flagellin-induced lysosomal pathway regulates inflammasome-dependent and -independent macrophage responses. Proc Natl Acad Sci U S A (2013) 110:E3321–30 10.1073/pnas.1305316110 23942123 PMC3761566

[83] Magalhaes JG Sorbara MT Girardin SE Philpott DJ What is new with nods? Curr Opin Immunol (2011) 23:29–34 10.1016/j.coi.2010.12.003 21190821

[84] Arnoult D Soares F Tattoli I Castanier C Philpott DJ Girardin SE An N-terminal addressing sequence targets NLRX1 to the mitochondrial matrix. J Cell Sci (2009) 122:3161–8 10.1242/jcs.051193 19692591 PMC2871076

[85] Moore CB Bergstralh DT Duncan JA Lei Y Morrison TE Zimmermann AG NLRX1 is a regulator of mitochondrial antiviral immunity. Nature (2008) 451:573–7 10.1038/nature06501 18200010

[86] Tattoli I Sorbara MT Vuckovic D Ling A Soares F Carneiro LAM Amino acid starvation induced by invasive bacterial pathogens triggers an innate host defense program. Cell Host Microbe (2012) 11:563–75 10.1016/j.chom.2012.04.012 22704617

[87] Eibl C Grigoriu S Hessenberger M Wenger J Puehringer S Pinheiro AS Structural and functional analysis of the NLRP4 pyrin domain. Biochemistry (2012) 51:7330–41 10.1021/bi3007059 22928810 PMC3445046

[88] Cui J Li Y Zhu L Liu D Songyang Z Wang HY NLRP4 negatively regulates type I interferon signaling by targeting the kinase TBK1 for degradation via the ubiquitin ligase DTX4. Nat Immunol (2012) 13:387–95 10.1038/ni.2239 22388039 PMC3760161

[89] Klionsky DJ Autophagy: from phenomenology to molecular understanding in less than a decade. Nat Rev Mol Cell Biol (2007) 8:931–7 10.1038/nrm2245 17712358

[90] Yang Z Klionsky DJ Eaten alive: a history of macroautophagy. Nat Cell Biol (2010) 12:814–22 10.1038/ncb0910-814 20811353 PMC3616322

[91] Choi AMK Ryter SW Levine B Autophagy in human health and disease. N Engl J Med (2013) 368:651–62 10.1056/NEJMra1205406 23406030

[92] Hussey S Travassos LH Jones NL Autophagy as an emerging dimension to adaptive and innate immunity. Semin Immunol (2009) 21:233–41 10.1016/j.smim.2009.05.004 19502083 PMC7129798

[93] Mizushima N Levine B Cuervo AM Klionsky DJ Autophagy fights disease through cellular self-digestion. Nature (2008) 451:1069–75 10.1038/nature06639 18305538 PMC2670399

[94] Besteiro S Brooks CF Striepen B Dubremetz JF Autophagy protein Atg3 is essential for maintaining mitochondrial integrity and for normal intracellular development of *Toxoplasma gondii* tachyzoites. PLoS Pathog (2011) 7(12):e1002416 10.1371/journal.ppat.1002416 22144900 PMC3228817

[95] Klionsky DJ Abdalla FC Abeliovich H Abraham RT Acevedo-Arozena A Adeli K Guidelines for the use and interpretation of assays for monitoring autophagy. Autophagy (2012) 8:445–544 10.4161/auto.19926 22966490 PMC3404883

[96] Ragusa MJ Stanley RE Hurley JH Architecture of the Atg17 complex as a scaffold for autophagosome biogenesis. Cell (2012) 151:1501–12 10.1016/j.cell.2012.11.028 23219485 PMC3806636

[97] Alers S Löffler AS Paasch F Dieterle AM Keppeler H Lauber K Atg13 and FIP200 act independently of Ulk1 and Ulk2 in autophagy induction. Autophagy (2011) 7:1423–33 10.4161/auto.7.12.18027 22024743 PMC3327613

[98] Chang T-K Shravage BV Hayes SD Powers CM Simin RT Wade Harper J Uba1 functions in Atg7- and Atg3-independent autophagy. Nat Cell Biol (2013) 15:1067–78 10.1038/ncb2804 23873149 PMC3762904

[99] Nishida Y Arakawa S Fujitani K Yamaguchi H Mizuta T Kanaseki T Discovery of Atg5/Atg7-independent alternative macroautophagy. Nature (2009) 461:654–8 10.1038/nature08455 19794493

[100] Kunz J Henriquez R Schneider U Deuter-Reinhard M Movva NR Hall MN Target of rapamycin in yeast, TOR2, is an essential phosphatidylinositol kinase homolog required for G1 progression. Cell (1993) 73:585–96 10.1016/0092-8674(93)90144-F 8387896

[101] Schu PV Takegawa K Fry MJ Stack JH Waterfield MD Emr SD Phosphatidylinositol 3-kinase encoded by yeast VPS34 gene essential for protein sorting. Science (1993) 260:88–91 10.1126/science.8385367 8385367

[102] Brunn GJ Williams J Sabers C Wiederrecht G Lawrence JC Abraham RT Direct inhibition of the signaling functions of the mammalian target of rapamycin by the phosphoinositide 3-kinase inhibitors, wortmannin and LY294002. EMBO J (1996) 15:5256–67 8895571 PMC452270

[103] Fruman DA Towards an understanding of isoform specificity in phosphoinositide 3-kinase signalling in lymphocytes. Biochem Soc Trans (2004) 32:315–9 10.1042/BST0320315 15046598

[104] Alessi DR Andjelkovic M Caudwell B Cron P Morrice N Cohen P Mechanism of activation of protein kinase B by insulin and IGF-1. EMBO J (1996) 15:6541–51 8978681 PMC452479

[105] Díaz-Troya S Pérez-Pérez ME Florencio FJ Crespo JL The role of TOR in autophagy regulation from yeast to plants and mammals. Autophagy (2008) 4:851–65 18670193 10.4161/auto.6555

[106] Hay N Upstream and downstream of mTOR. Genes Dev (2004) 18:1926–45 10.1101/gad.1212704 15314020

[107] Takeuchi H Kondo Y Fujiwara K Kanzawa T Aoki H Mills GB Synergistic augmentation of rapamycin-induced autophagy in malignant glioma cells by phosphatidylinositol 3-kinase/protein kinase B inhibitors. Cancer Res (2005) 65:3336–46 10.1158/0008-5472.CAN-04-3640 15833867

[108] Jung CH Jun CB Ro SH Kim YM Otto NM Cao J ULK-Atg13-FIP200 complexes mediate mTOR signaling to the autophagy machinery. Mol Biol Cell (2009) 20:1992–2003 10.1091/mbc.E08-12-1249 19225151 PMC2663920

[109] Kraft C Kijanska M Kalie E Siergiejuk E Lee SS Semplicio G Binding of the Atg1/ULK1 kinase to the ubiquitin-like protein Atg8 regulates autophagy. EMBO J (2012) 31:3691–703 10.1038/emboj.2012.225 22885598 PMC3442273

[110] Linares JF Duran A Yajima T Pasparakis M Moscat J Diaz-Meco MT K63 polyubiquitination and activation of mTOR by the p62-TRAF6 complex in nutrient-activated cells. Mol Cell (2013) 51:283–96 10.1016/j.molcel.2013.06.020 23911927 PMC3971544

[111] Lindmo K Regulation of membrane traffic by phosphoinositide 3-kinases. J Cell Sci (2006) 119:605–14 10.1242/jcs.02855 16467569

[112] Petiot A Ogier-Denis E Blommaart EF Meijer AJ Codogno P Distinct classes of phosphatidylinositol 3’-kinases are involved in signaling pathways that control macroautophagy in HT-29 cells. J Biol Chem (2000) 275:992–8 10.1074/jbc.275.2.992 10625637

[113] Cebollero E van der Vaart A Zhao M Rieter E Klionsky DJ Helms JB Phosphatidylinositol-3-phosphate clearance plays a key role in autophagosome completion. Curr Biol (2012) 22:1545–53 10.1016/j.cub.2012.06.029 22771041 PMC3615650

[114] Boya P Reggiori F Codogno P Emerging regulation and functions of autophagy. Nat Cell Biol (2013) 15:713–20 10.1038/ncb2788 23817233 PMC7097732

[115] Yuk JM Yoshimori T Jo E-K Autophagy and bacterial infectious diseases. Exp Mol Med (2012) 44:99–108 10.3858/emm.2012.44.2.032 22257885 PMC3296818

[116] Nakagawa I Amano A Mizushima N Yamamoto A Yamaguchi H Kamimoto T Autophagy defends cells against invading group A *Streptococcus*. Science (2004) 306:1037–40 10.1126/science.1103966 15528445

[117] Kayath CA Hussey S El hajjami N Nagra K Philpott D Allaoui A Escape of intracellular *Shigella* from autophagy requires binding to cholesterol through the type III effector, IcsB. Microbes Infect (2010) 12:956–66 10.1016/j.micinf.2010.06.006 20599519

[118] Ogawa M Yoshimori T Suzuki T Sagara H Mizushima N Sasakawa C Escape of intracellular *Shigella* from autophagy. Science (2005) 307:727–31 10.1126/science.1106036 15576571

[119] Dupont N Lacas-Gervais S Bertout J Paz I Freche B Van Nhieu GT *Shigella* phagocytic vacuolar membrane remnants participate in the cellular response to pathogen invasion and are regulated by autophagy. Cell Host Microbe (2009) 6:137–49 10.1016/j.chom.2009.07.005 19683680

[120] Cullinane M Gong L Li X Lazar-Adler N Tra T Wolvetang E Stimulation of autophagy suppresses the intracellular survival of *Burkholderia pseudomallei* in mammalian cell lines. Autophagy (2008) 4:744–53 18483470 10.4161/auto.6246

[121] Birmingham CL Canadien V Kaniuk NA Steinberg BE Higgins DE Brumell JH Listeriolysin O allows *Listeria monocytogenes* replication in macrophage vacuoles. Nature (2008) 451:350–4 10.1038/nature06479 18202661

[122] Birmingham CL Autophagy controls *Salmonella* infection in response to damage to the *Salmonella*-containing vacuole. J Biol Chem (2006) 281:11374–83 10.1074/jbc.M509157200 16495224

[123] Gutierrez MG Master SS Singh SB Taylor GA Colombo MI Deretic V Autophagy is a defense mechanism inhibiting BCG and *Mycobacterium tuberculosis* survival in infected macrophages. Cell (2004) 119:1–14 10.1016/j.cell.2004.11.038 15607973

[124] Orvedahl A Alexander D Tallóczy Z Sun Q Wei Y Zhang W HSV-1 ICP34.5 confers neurovirulence by targeting the Beclin 1 autophagy protein. Cell Host Microbe (2007) 1:23–35 10.1016/j.chom.2006.12.001 18005679

[125] Lee HK Lund JM Ramanathan B Mizushima N Iwasaki A Autophagy-dependent viral recognition by plasmacytoid dendritic cells. Science (2007) 315:1398–401 10.1126/science.1136880 17272685

[126] Xu Y Jagannath C Liu X-D Sharafkhaneh A Kolodziejska KE Eissa NT Toll-like receptor 4 is a sensor for autophagy associated with innate immunity. Immunity (2007) 27:135–44 10.1016/j.immuni.2007.05.022 17658277 PMC2680670

[127] Delgado MA Elmaoued RA Davis AS Kyei G Deretic V Toll-like receptors control autophagy. EMBO J (2008) 27:1110–21 10.1038/emboj.2008.31 18337753 PMC2323261

[128] Shi C-S Kehrl JH MyD88 and Trif target Beclin 1 to trigger autophagy in macrophages. J Biol Chem (2008) 283:33175–82 10.1074/jbc.M804478200 18772134 PMC2586260

[129] Schmitz F Heit A Dreher S Eisenächer K Mages J Haas T Mammalian target of rapamycin (mTOR) orchestrates the defense program of innate immune cells. Eur J Immunol (2008) 38:2981–92 10.1002/eji.200838761 18924132

[130] Yano T Mita S Ohmori H Oshima Y Fujimoto Y Ueda R Autophagic control of listeria through intracellular innate immune recognition in *Drosophila*. Nat Immunol (2008) 9:908–16 10.1038/ni.1634 18604211 PMC2562576

[131] Cooney R Baker J Brain O Danis B Pichulik T Allan P NOD2 stimulation induces autophagy in dendritic cells influencing bacterial handling and antigen presentation. Nat Med (2009) 16:90–7 10.1038/nm.2069 19966812

[132] Travassos LH Carneiro LAM Ramjeet M Hussey S Kim Y-G Magalhaes JG Nod1 and Nod2 direct autophagy by recruiting ATG16L1 to the plasma membrane at the site of bacterial entry. Nat Immunol (2010) 11:55–62 10.1038/ni.1823 19898471

[133] Rioux JD Xavier RJ Taylor KD Silverberg MS Goyette P Huett A Genome-wide association study identifies new susceptibility loci for Crohn disease and implicates autophagy in disease pathogenesis. Nat Genet (2007) 39:596–604 10.1038/ng2032 17435756 PMC2757939

[134] Kuballa P Huett A Rioux JD Daly MJ Xavier RJ Impaired autophagy of an intracellular pathogen induced by a Crohn’s disease associated ATG16L1 variant. PLoS One (2008) 3:e3391 10.1371/journal.pone.0003391 18852889 PMC2566595

[135] Glubb DM Gearry RB Barclay ML Roberts RL Pearson J Keenan JI NOD2 and ATG16L1 polymorphisms affect monocyte responses in Crohn’s disease. World J Gastroenterol (2011) 17:2829–37 10.3748/wjg.v17.i23.2829 21734790 PMC3120942

[136] Plantinga TS Joosten LA Netea MG ATG16L1 polymorphisms are associated with NOD2-induced hyperinflammation. Autophagy (2011) 7:1074–5 10.4161/auto.7.9.15867 21673517

[137] Kleinnijenhuis J Oosting M Plantinga TS van der Meer JWM Joosten LAB Crevel RV Autophagy modulates the *Mycobacterium tuberculosis*-induced cytokine response. Immunology (2011) 134:341–8 10.1111/j.1365-2567.2011.03494.x 21978003 PMC3209573

[138] Byrne BG Dubuisson JF Joshi AD Persson JJ Swanson MS Inflammasome components coordinate autophagy and pyroptosis as macrophage responses to infection. MBio (2012) 4:e620–612 10.1128/mBio.00620-12 23404401 PMC3573666

[139] Lei Y Wen H Yu Y Taxman DJ Zhang L Widman DG The mitochondrial proteins NLRX1 and TUFM form a complex that regulates Type I interferon and autophagy. Immunity (2012) 36:933–46 10.1016/j.immuni.2012.03.025 22749352 PMC3397828

[140] Shelly S Lukinova N Bambina S Berman A Cherry S Autophagy is an essential component of *Drosophila* immunity against vesicular stomatitis virus. Immunity (2009) 30:588–98 10.1016/j.immuni.2009.02.009 19362021 PMC2754303

[141] Soares F Tattoli I Wortzman ME Arnoult D Philpott DJ Girardin SE NLRX1 does not inhibit MAVS-dependent antiviral signalling. Innate Immun (2012) 19(4):438–48 10.1177/1753425912467383 23212541

[142] Rebsamen M Vazquez J Tardivel A Guarda G Curran J Tschopp J NLRX1/NOD5 deficiency does not affect MAVS signalling. Cell Death Differ (2011) 18:1387–1387 10.1038/cdd.2011.64 21617692 PMC3172102

[143] Allen IC Wilson JE Schneider M Lich JD Roberts RA Arthur JC NLRP12 suppresses colon inflammation and tumorigenesis through the negative regulation of noncanonical NF-κB signaling. Immunity (2012) 36:742–54 10.1016/j.immuni.2012.03.012 22503542 PMC3658309

[144] Jounai N Kobiyama K Shiina M Ogata K Ishii KJ Takeshita F NLRP4 negatively regulates autophagic processes through an association with Beclin1. J Immunol (2011) 186:1646–55 10.4049/jimmunol.1001654 21209283

[145] Levine B Mizushima N Virgin HW Autophagy in immunity and inflammation. Nature (2011) 469:323–35 10.1038/nature09782 21248839 PMC3131688

[146] Jounai N Takeshita F Kobiyama K Sawano A Miyawaki A Xin KQ The Atg5 Atg12 conjugate associates with innate antiviral immune responses. Proc Natl Acad Sci U S A (2007) 104:14050–5 10.1073/pnas.0704014104 17709747 PMC1955809

[147] Tal MC Sasai M Lee HK Yordy B Shadel GS Iwasaki A Absence of autophagy results in reactive oxygen species-dependent amplification of RLR signaling. Proc Natl Acad Sci U S A (2009) 106:2770–5 10.1073/pnas.0807694106 19196953 PMC2650341

[148] Lupfer C Thomas PG Anand PK Vogel P Milasta S Martinez J Receptor interacting protein kinase 2–mediated mitophagy regulates inflammasome activation during virus infection. Nat Immunol (2013) 14:480–8 10.1038/ni.2563 23525089 PMC3631456

[149] Harris J Hartman M Roche C Zeng SG O’Shea A Sharp FA Autophagy controls IL-1 secretion by targeting pro-IL-1 for degradation. J Biol Chem (2011) 286:9587–97 10.1074/jbc.M110.202911 21228274 PMC3058966

[150] Saitoh T Fujita N Jang MH Uematsu S Yang B-G Satoh T Loss of the autophagy protein Atg16L1 enhances endotoxin-induced IL-1β production. Nature (2008) 456:264–8 10.1038/nature07383 18849965

[151] Shi C-S Shenderov K Huang N-N Kabat J Abu-Asab M Fitzgerald KA Activation of autophagy by inflammatory signals limits IL-1β production by targeting ubiquitinated inflammasomes for destruction. Nat Immunol (2012) 13:255–63 10.1038/ni.2215 22286270 PMC4116819

[152] Bauernfeind F Bartok E Rieger A Franchi L Nuñez G Hornung V Cutting edge: reactive oxygen species inhibitors block priming, but not activation, of the NLRP3 inflammasome. J Immunol (2011) 187:613–7 10.4049/jimmunol.1100613 21677136 PMC3131480

[153] Dostert C Pétrilli V van Bruggen R Steele C Mossman BT Tschopp J Innate immune activation through Nalp3 inflammasome sensing of asbestos and silica. Science (2008) 320:674–7 10.1126/science.1156995 18403674 PMC2396588

[154] Latz E NOX-free inflammasome activation. Blood (2010) 116:1393–4 10.1182/blood-2010-06-287342 20813905

[155] Meissner F Seger RA Moshous D Fischer A Reichenbach J Zychlinsky A Inflammasome activation in NADPH oxidase defective mononuclear phagocytes from patients with chronic granulomatous disease. Blood (2010) 116:1570–3 10.1182/blood-2010-01-264218 20495074 PMC2938844

[156] Davis BK Wen H Ting JP The inflammasome NLRs in immunity, inflammation, and associated diseases. Annu Rev Immunol (2011) 29:707–35 10.1146/annurev-immunol-031210-101405 21219188 PMC4067317

[157] Dupont N Jiang S Pilli M Ornatowski W Bhattacharya D Deretic V Autophagy-based unconventional secretory pathway for extracellular delivery of IL-1β. EMBO J (2011) 30:4701–11 10.1038/emboj.2011.398 22068051 PMC3243609

[158] Juliana C Fernandes-Alnemri T Kang S Farias A Qin F Alnemri ES Non-transcriptional priming and deubiquitination regulate NLRP3 inflammasome activation. J Biol Chem (2012) 287:36617–22 10.1074/jbc.M112.407130 22948162 PMC3476327

[159] Girardin SE Travassos LH Hervé M Blanot D Boneca IG Philpott DJ Peptidoglycan molecular requirements allowing detection by Nod1 and Nod2. J Biol Chem (2003) 278:41702–8 10.1074/jbc.M307198200 12871942

